# Syntactic learning by mere exposure - An ERP study in adult learners

**DOI:** 10.1186/1471-2202-10-89

**Published:** 2009-07-29

**Authors:** Jutta L Mueller, Regine Oberecker, Angela D Friederici

**Affiliations:** 1 Max Planck Institute for Human Cognitive and Brain Science, Stephanstr. 1a, 04103 Leipzig, Germany

## Abstract

**Background:**

Artificial language studies have revealed the remarkable ability of humans to extract syntactic structures from a continuous sound stream by mere exposure. However, it remains unclear whether the processes acquired in such tasks are comparable to those applied during normal language processing. The present study compares the ERPs to auditory processing of simple Italian sentences in native and non-native speakers after brief exposure to Italian sentences of a similar structure. The sentences contained a non-adjacent dependency between an auxiliary and the morphologically marked suffix of the verb. Participants were presented four alternating learning and testing phases. During learning phases only correct sentences were presented while during testing phases 50 percent of the sentences contained a grammatical violation.

**Results:**

The non-native speakers successfully learned the dependency and displayed an N400-like negativity and a subsequent anteriorily distributed positivity in response to rule violations. The native Italian group showed an N400 followed by a P600 effect.

**Conclusion:**

The presence of the P600 suggests that native speakers applied a grammatical rule. In contrast, non-native speakers appeared to use a lexical form-based processing strategy. Thus, the processing mechanisms acquired in the language learning task were only partly comparable to those applied by competent native speakers.

## Background

Language is a highly structured temporally sequenced stream of vocal sounds. One of the first steps in learning a language is to discover and to analyze its sequential structure which is by no means a trivial task. Leaving aside the acquisition of phonological and semantic aspects language acquisition can be seen, at least in part, as a special form of auditory sequence learning. This idea is supported, for example, by similar patterns of impairment in sequence learning and language tasks in aphasia, computer simulation studies, and artificial grammar learning experiments with healthy participants which suggest at least partially shared cognitive mechanisms in both auditory sequence and language learning [1-3]. Due to this close relationship sequence learning studies with artificial languages have been taken to model word or rule acquisition processes that operate in natural language learning. In these studies it has been shown that humans possess the ability to extract words as well as simple syntactic rules from an auditory input stream consisting of syllables or words [4,5]. It is important to mention that no explicit instruction of the underlying rule seems to be necessary in order to achieve this and that the learning process can be initiated by mere exposure to speech.

A relatively simple but important model for syntax acquisition in language has been non-adjacent dependency learning [6-11]. Natural language syntax is characterized not only by local phrase structure but also by dependency relations between more distant elements (e.g. subject-verb agreement: **The baby **(singular) who is in the bed **is **(singular) laughing, or auxiliary-main verb inflection agreement: The baby **is **laugh**ing**.). Such dependencies finally enable the construction of hierarchical sentence structures which are considered defining features of human language [12]. Being able to learn and process distant relations between linguistic elements is thus a basic prerequisite for acquiring syntax. There are a number of behavioural studies which have demonstrated the human ability to learn non-adjacent dependencies by mere exposure to artificial or natural speech [6-9,13,14]. In the literature artificial grammar learning studies can be found in different research areas. Depending on the learning process which is proposed by the researchers they are referred to as statistical learning, rule learning or incidental learning studies although they often investigate the learning of similar structures. Here, we prefer to use unbiased terminology and refer to our paradigm as 'learning by mere exposure'.

In non-adjacent dependency learning studies structures of the form AXC are used whereby A reliably predicts C while the X element is variable (henceforth, AXC structures are also referred to as triplets). The relevant units can be either pseudowords [6,7], syllables [8,9], or segments [15,16]. The learnability of AXC structures seems to strongly depend on the presence of additional cues which can support the structuring of the incoming information. At least minimal pre-segmentation of the triplets, for example, seems to be a prerequisite for the acquisition and generalization of a non-adjacent rule [8]. The type of segment carrying the dependency (syllables, consonants, vowels) seems to also play a role [9,15,16].

Likewise, a certain degree of variability of the intervening X elements appears to be necessary in order to detect the predictive relation between A and C [6]. Given these prerequisites, learning of non-adjacent dependencies is mastered in present artificial grammar settings even by infants starting from the age of 15 months [6,7,14]. However, there is considerable disagreement concerning the learning mechanism which is responsible for the acquisition of non-adjacent dependencies and the representational format of the acquired knowledge. Proponents of a dual learning mechanism assume a statistical learning mechanism that draws upon distributional information on the one hand and a separate rule-learning mechanism which operates on a faster, possibly all-or-nothing basis on the other [8,17,18]. While the first mechanism is statistical in nature, the latter is assumed to be relatively independent of frequency information [8]. In contrast, advocates of a unitary account argue that language learning in general is based on a statistical learning process using distributional information [19-21]. This debate is reminiscent of another long-standing controversy in artificial grammar learning with visually presented finite-state grammars of the Reber type [22]. While some researchers argue that the type of knowledge which is acquired can be described as an abstract rule [22], others claim that what is learned is more stimulus-specific and related to surface features of the grammar [23,24]. Importantly, this does not mean that every possible statistic is calculated - this would lead to a computational explosion. Rather, the system is thought to pick out relevant features for distributional analysis.

The present study set out to tap into this debate from an adult language-learning perspective. Rather than trying to differentiate between statistical and rule-guided learning within the artificial grammar framework, this study was designed to look at the outcome of syntactic learning in contrast to established, native syntactic processes in a natural language. Native speakers have a highly generalized knowledge of the syntactic constructions of their language and thus can be assumed to use syntactic rules. A recent study on syntactic processing in native adults indeed demonstrates that they behave according to syntactic rules rather than to distributional probabilities of related elements [25]. Therefore the question arises whether the rule-like behaviour acquired in classical non-adjacent dependency learning tasks is comparable to the processes applied during normal language syntactic processing. If a rule processing mechanism is available to learners from the beginning or after a short learning phase the processing of non-adjacent dependency learning tasks should share characteristic features with natural language processing. If, however, no rule processing mechanism (and instead a statistical learning process) is used by learners, non-adjacent dependency processing in learners should be different from that of native-speaking adults.

In order to directly contrast non-adjacent dependency processing as acquired under different learning conditions ('artificial' vs. natural) we used Italian language stimuli in a typical artificial grammar learning design. For Italian native speakers, the sentences were thus part of their native language, and for learners the same stimuli were a semantic-free miniature language which, for these naive participants, is not any different to an invented artificial grammar. Thus, the present experiment can be viewed as a test of the degree of similarity between the processing of non-adjacent dependencies in artificial and native language.

We chose to use event-related-potentials (ERPs) because they allowed us to distinguish different types and stages of cognitive and linguistic processing with a very high time-resolution. There are numerous ERP studies investigating syntactic processes in natural language involving non-adjacent dependencies. A prototypical case for example is morphological agreement marking. Violations of morphosyntactic agreement (e.g. for number, tense, gender) regularly result in an ERP pattern consisting of a left anterior negativity (LAN) and/or a posterior positivity (P600) [26-31]. In this context, the LAN is usually seen as indicating relatively automatic (morpho)syntactic processes while the P600 is thought to represent later and more controlled syntactic processes. With respect to artificial grammar learning, in particular, it might be interesting to look at respective studies with second language (L2) learners. Some studies with L2 speakers have shown that the P600 develops with high proficiency in an L2 [32-35]. Other L2 speakers show no P600 effect [36,37] or an N400 in initial stages of learning instead [34]. The N400 is a negative wave which is normally elicited by lexico-semantic manipulations and is thus, seen as reflecting semantic integration [38] or lexical access [39]. The finding of an N400 effect instead of the expected P600 was taken to reflect a lexical processing strategy. It is assumed that L2 speakers probably memorized full forms in the initial stages of learning whereas more proficient L2 speakers and native speakers use their grammatical knowledge to de-compose morphologically complex forms [34]. A native-like LAN component for morphosyntactic violations is seen only for very proficient L2 speakers [35,40].

With respect to artificial grammar studies, it was shown that the P600 as well as anterior negativities could be elicited in syntactic violation conditions after short training periods [33,41,42]. In all of these studies participants were trained in a very explicit manner with plenty of feedback. There are only two ERP studies on non-adjacent dependency processing which used a mere exposure design. De Diego Balaguer and colleagues [11] presented participants with an AXC language consisting of trisyllabic 'words' which were pre-segmented by 25 ms pauses. In the data analysis the authors focused on the learning phases and found indication that word learning and rule extraction might be reflected by two different ERPs. While word learning led to an increase of the N400 effect, rule learning led to an increase of the P2 effect during the learning phase. As no rule violations were presented in the test phases in this study, it is impossible to know whether anterior negativities and/or P600 would have been elicited. A study by Mueller, Bahlmann and Friederici [10] investigated non-adjacent dependency learning between bisyllabic non-words. Processing of non-adjacent dependencies was tested using violations of the AXC structure by introducing an incorrect element at the final position (AXX). An anterior negativity and a subsequent positivity were found to accompany the ability to discriminate between correct and incorrect structures.

However, from this study it is difficult to conclude whether non-adjacent dependencies were learned or just a local rule (as the test condition was a local violation of the expected item category). Second, as in the study of De Diego Balaguer et al. [11], the stimuli were taken from a non-existent artificial language, which furthermore, was created with a speech synthesizer. This precludes a direct comparison of these studies to non-adjacent dependency processing in natural language.

The present study aimed to fill this gap and to directly contrast the outcome of natural language learning (looking at adult native language processing) with the outcome of non-adjacent dependency learning by mere exposure (looking at adult L2 processing). It is not difficult to think of quite a few principle distinctions between artificial (L2) grammar learning and natural language processing. While first language acquisition is a process extending over the first years of life, experimental simulations of this process, in the form of incidental language learning tasks, have observed the acquisition of structural knowledge from language-like acoustic input within a time scale of minutes. In artificial language studies the learning phase usually contains a concatenated string of a very small variety of words or phrases, a situation which would be absurd in real life in which the stimuli to be learned never occur with comparable frequency over such a short time period. It is quite conceivable that these differences have some impact on the degree of similarity and dissimilarity of cognitive operations during non-adjacent dependency processing in native language learning and incidental adult artificial (L2) grammar learning. One outcome, for example, could be that the massed exposure and the uniform stimuli result in a more item-based knowledge which is less generalized and abstract compared to native language knowledge. The contrary could also be the case, namely that the high level of exposure in a short time results in the very fast extraction of the rule and thus in very skilled, native-like processing.

The stimulus material in our study comprised simple Italian sentences that contained a non-adjacent dependency between an auxiliary and a main verb's suffix. These structures are natural language equivalents of stimuli used in previous non-adjacent dependency learning studies [6,8]. Our experiment comprised four alternate learning and testing phases. During learning phases, participants listened to correct sentences only. During testing phases, participants were exposed to correct and incorrect examples and had to give grammaticality judgments on each stimulus. No feedback was given. Examples of the stimulus material can be seen in Table 1.

**Table 1:** Examples from the stimulus material. Incorrect sentences are marked with asterisks.

	auxiliary 'sta'	auxiliary 'può'
correct	(1) Il fratello sta arrivando.	(2) Il fratello può arrivare.
	*'The brother is arriving'*.	*'The brother can arrive'*.

incorrect	(3) *Il fratello sta arrivare.	(4) *Il fratello può arrivando.

We hypothesized that native speakers of Italian would display an anterior negativity and/or a P600 effect, as frequently reported for morphosyntactic violations in natural languages. If adult incidental artificial (L2) grammar learning is similar to native language learning, learners would demonstrate the full 'native' pattern. Alternatively, if the two learning conditions result in dissimilar cognitive operations, only parts of the native pattern, or a qualitatively different ERP pattern (e.g. an N400 instead of a P600) should be observable. The latter would be taken to indicate that a native-like syntactic operation is not yet established possibly suggesting that no general syntactic rule is extracted at this stage of learning.

## Results and Discussion

### Results

**Figure 1:**
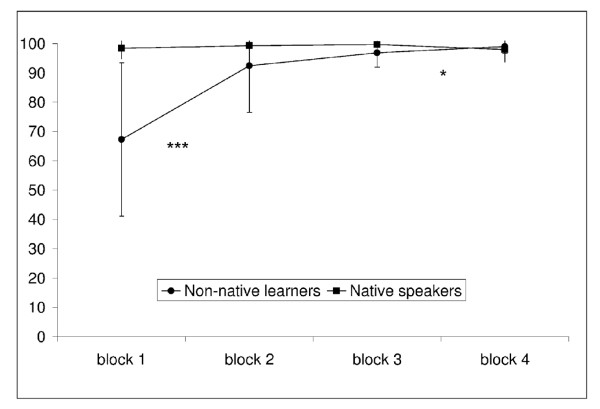
**Performance rates**. This figure illustrates performance rates for each group of participants in each testing block. The y-axis represents the percentage of correct responses. The asterisks stand for the statistical significance of the difference between two subsequent testing blocks (*** = *p *< .0001, * = *p *< .05).

The behavioural results for native speakers were 99% correct answers (SD 2%) in the grammaticality judgment task. Learners had 89% correct answers (SD 9%). Figure 1 shows the performance rates for each group of participants across testing blocks. In order to check if there was an ongoing learning process from the first to the fourth learning phase we tested the performance rates across the different testing blocks in two separate ANOVAs with the repeated measures factor BLOCK (testing block 1 to 4) for each group.

There was a significant main effect of BLOCK for the non-native learners [F(3,87) = 29.75, p < 0.0001]. Further t-tests for simple main effects revealed significant differences between the first and the second [t(29) = -5.24, p < .0001] and the third and the fourth block [t(29) = -2.28, p = .030]. There was no significant effect of the factor BLOCK for the native speakers.

**Figure 2:**
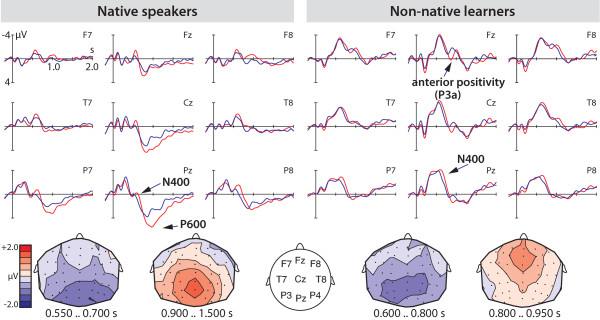
**ERP results**. ERPs of correctly (blue lines) and incorrectly suffixed (red lines) verbs in native speakers of Italian and non-native learners. The legend including the definition of the time and amplitude axis is displayed at the upper left panel (electrode F7). The isovoltage maps show the scalp distributions of the difference potentials between incorrectly and correctly suffixed verbs. The legend showing the colourcoding of the amplitudes in the isovoltage maps is displayed at the left side of the scalp images.

Due to the relatively low number of trials in each of the separate testing blocks (8 trials per condition), we did not analyse the ERPs of each block separately. However, to reduce effects of incorrectly processed items, we only used correctly answered trials for the ERP analysis. The ERPs for the native Italian group displayed a centro-parietally distributed negativity and a subsequent similarly distributed positivity (see Figure 2). Hence, two time windows were chosen for analysis. In the first time window from 550 to 700 ms after the onset of the verb the factor CORR (comprising correctly vs. incorrectly inflected verbs) reached significance [F(1,18) = 5.77, p = 0.027, *ω*^2 ^= 0.025] over lateral sites. The mean amplitudes on the P8 electrode were -1.37 *μ*V (SE 0.53) for the incorrect condition and -0.05 *μ*V (SE 0.43) for the correct one.

For the midline electrodes there was no significant effect of CORR. For the second time window between 900 and 1500 ms there was a significant interaction of the factors CORR and REG (comprising anterior vs. posterior brain regions) [F(1,18) = 7.40, p = 0.014, *ω*^2 ^= 0.015]. Further analyses for each region separately revealed no simple main effect of CORR over anterior electrode sites, but a significant simple main effect of CORR over posterior sites [F(1,18) = 5.72, p = 0.028, *ω*^2 ^= 0.022]. The mean amplitudes on the P8 electrode were 1.84 *μ*V (SE 0.35) for the incorrect condition and 1.23 *μ*V (SE 0.23) for the correct one. For the midline electrodes there was a significant main effect of CORR [F(1,18) = 4.72, p = 0.044, *ω*^2 ^= 0.018] and an interaction of CORR by REG [F(1,18) = 8.66, p = 0.009, *ω*^2 ^= 0.018]. Subsequent analyses revealed a significant simple main effect of CORR over the posterior midline [F(1,18) = 10.88, p = 0.004, *ω*^2 ^= 0.047]. The mean amplitudes on the Pz electrode were 3.67 *μ*V (SE 0.37) for the incorrect condition and 1.85 *μ*V (SE 0.37) for the correct one.

The non-native learners also displayed a centro-parietally distributed negativity and a subsequent positivity which, in contrast to the native speakers, was anteriorily distributed (see Figure 2). In the first time window from 600 to 800 ms after verb onset there was a main effect of the factor CORR [F(1,29) = 5.13, p = 0.031, *ω*^2 ^= 0.02]. Over midline electrode sites the factor CORR did not lead to a significant effect. The mean amplitudes on the P8 electrode were -1.77 *μ*V (SE 0.26) for the incorrect condition and -1.32 *μ*V (SE 0.23) for the correct one. For the second time window from 800 to 950 ms, there were no significant effects over lateral electrode sites. Over midline electrodes there was a main effect of CORR [F(1,29) = 4.44, p = 0.044, *ω*^2 ^= 0.017] and a marginally significant interaction of CORR and REG [F(1,29) = 3.34, p = 0.078, *ω*^2 ^= 0.006]. Subsequent tests revealed a significant effect of CORR over anterior midline sites [F(1,29) = 10.14, p = 0.004, *ω*^2 ^= 0.044] while there was no significant effect over posterior midline electrode sites. The mean amplitudes on the Fz electrode were -0.82 *μ*V (SE 0.31) for the incorrect condition and -1.52 *μ*V (SE 0.30) for the correct one.

In sum, the statistical analyses confirmed a centro-parietally distributed negativity for both the native speakers and the non-native learners. Both groups showed a positivity which was, however, different in timing and distribution.

## Discussion

The present study aimed to investigate the neurocognitive processes acquired in standard incidental grammar learning tasks by comparing them to syntactic processes in natural language. We recorded ERPs while participants listened to grammatical and ungrammatical sentences in a pseudo-artificial language.

This language was a subset of Italian thus enabling us to compare the processing of grammatical and ungrammatical sequences in learners and in speakers of the language who had learned the language in a native setting and had a lifetime of experience with it. This design allowed us to draw conclusions about the similarity of syntactic processes applied by native language users and rule-processing mechanisms acquired in adult grammar learning studies using a mere exposure procedure.

As expected, Italian native speakers showed almost perfect behavioural performance in the grammaticality judgment task of the Italian stimulus sentences. In the ERP they displayed a biphasic pattern of a centro-parietal negativity followed by a long-lasting positivity. Given the timing and distribution of these ERP effects they presumably represent an N400 and a subsequent P600 component. As the onset of the negativity seems to be quite late (550 ms), we measured the average length of the verb stems and the suffixes. The acoustic analyses revealed an average length of 259 ms (SD 61 ms) for the verb stems and an average length of 492 ms (SD 48) for the suffixes. This means that the observed negativity with a latency of 550 ms after verb onset indeed falls in the classic N400 time window.

At first sight the ERP response is surprising as the N400 is regarded as a lexical-semantic component and the P600 as a syntactic one. However, there is evidence that the N400 does not only occur for lexical processes at the level of semantic integration, but also for difficulties at the stage of lexical access [39]. The N400 has been shown in learning experiments in which the absence of any semantic meaning allowed researchers to relate the N400 to the access of lexical forms only [42-44]. Thus, it is conceivable that the present N400 for the Italian native speakers represents lexical difficulties when encountering an unexpected suffix. This leaves us with the question why no N400 effects are observed in other studies using agreement violation conditions. A potential explanation might be the highly repetitive design of the present study in which virtually every sentence contained the same grammatical structure and no filler items were used (because this would have disturbed the acquisition process for the learners). Even though the expectation of a specific suffix form in a specific grammatical construction is very high in any study investigating a grammatical rule and its violation the repetitive use of the same sentence structures in the present study may have led to a specific focus on the frequently reoccurring suffix forms. This circumstance might also explain why there was no left anterior negativity in the present study.

In contrast, the P600 observed for the native speakers can be taken as a direct reflection of syntactic difficulties brought about by the incorrect verb suffix. The P600 occurs in a wide range of conditions that include rule violations as in, for example, linguistic syntactic processing [26,27,29-31], abstract sequence processing [45], music syntactic processing [46], and mathematical processing [47]. In linguistic syntactic violation conditions the P600 is usually seen as indicating syntactic integration and repair [48]. Thus, we take this component to reflect abstract syntactic rule representations used by native speakers as the basis for the violation detection during sentence processing.

The learners in our study demonstrated very good performance levels although they were lower than those of native speakers. Their less-than-perfect performance and improvement across testing blocks indicate that the learners are still at a beginning stage of acquisition of the non-adjacent dependency rule. The ERP effects they displayed for incorrect vs. correct non-adjacent dependencies are different from those observed for the native speakers. Learners showed a significant centro-parietal negativity between 600 and 800 ms and a subsequent anterior positivity between 800 and 950 ms. As in native speakers the negativity is seen as an instance of an N400. The observation of the N400 in a slightly later time window compared to native speakers appears to reflect the fact that the lexical access processes are not yet as fast and efficient as in the native speakers even though the processes themselves are, in principle, identical. Delays in the N400 component in second language speakers are known from other studies [36,49] and thus the present result is perfectly in line with these. The positivity observed for the learners, however, is clearly different from the P600 effect observed for the native speakers, as it is present only over anterior midline electrode sites and is very brief. Given the morphology of this ERP effect, we speculate that the positivity belongs to the family of P300 components reflecting attention-related cognitive processes [50,51]. Specifically, the P3a would be a possible interpretation for the positivity in the present experiment. The P3a is characterized by a fronto-central distribution and is found in response to contextual novelty and taken as an indicator of a cognitive orienting response towards a novel stimulus. Importantly, the component is not thought to reflect the detection of the novel stimulus but rather the orientation towards that stimulus in order to evaluate it for further action. Although the participants in our study had already heard the suffixes -*ando *and -*are *in the learning phase they did not expect them to occur in the context of the respective incorrect auxiliary.

As the correctness of the suffix was task relevant it is conceivable that an orienting response, as reflected in the P3a component, took place. Against the background of this interpretation, we must assume attention-related processes to be in use specifically for the learners. However, regardless of whether this interpretation is correct the difference between the native and the non-native ERP pattern remains.

Learners do not show show any indication of abstract rule application similar to those observed in native speakers. This leads us to conclude that what the learners acquired in the present paradigm was a set of (phonological) expectations about specific stimulus forms but no abstract representation of a syntactic rule concerning the dependency between two elements.

The present data must be considered against the background of artificial grammar studies with quite complex grammars in which the participants reached a more native-like pattern [33,41,42]. In these studies, however, participants received an extensive amount of training including feedback. This might have been sufficient to induce syntactic rule-based processes similar to those applied by native speakers. Clearly, in the present learning paradigm of mere exposure, these were not available to the learners.

The present results support learning theories that assume a statistical learning mechanism rather than a rule-based extraction mechanism as an initial acquisition stage. If syntactic rules were acquired in an all-or-nothing rule extraction process, a P600 should have been present for the learners too. Instead, it seems to be the case that the learners use perceptual features of an item's form (N400) to guide acquisition and general cognitive attentional processes (P3a) in order to solve the grammaticality judgment task. This is consistent with a view of language learning in which the distributions of perceptual features are computed initially while generalized, abstracted knowledge is established in a later stage. A crucial issue for future research will be to describe the transition from the distributional to the rule-based stage during artificial and second language learning and also possibly in first language acquisition.

## Conclusion

The present study showed that syntactic processing mechanisms acquired in a non-adjacent dependency learning task without explicit feedback and instruction are different from syntactic processes applied during native language processing. Native speakers showed electrophysiological evidence of the application of a syntactic rule, whereas learners did not. The result speaks against a rule-extraction process on an all-or-nothing basis, at least for the present experimental paradigm. Rather before applying a generalized rule, the system probably uses lexically based expectations that are still closely linked to specific surface features (i.e. specific phonological realizations).

## Methods

### Participants

38 native speakers of German (18 female; age: range 18-29, mean 24) with no knowledge of Italian and 19 Italian native speakers (13 female; age: range 20-33, mean 25) participated in the study. All participants were right-handed and had no hearing or neurological disorders. All gave written informed consent in accordance with the declaration of Helsinki prior to the experiments. The study was approved by the ethics committee of the medical department at the University of Leipzig. Three of the German participants had to be excluded due to high artifact rates in the EEG, and 5 participants because of the absence of behavioural learning effects.

### Stimuli

The miniature version of Italian used in the study consisted of 2 articles (*il*, masculine definite article; *la*, feminine definite article), 2 animate nouns (*fratello*, brother; *sorella*, sister), two auxiliaries (*può*, to be able to, first person singular; *sta*, to be, first person singular) and 32 verbs which could occur in infinitive (e.g. *arrivare*) or in gerund form (e.g. *arrivando*). The complete list of verbs can be found in Additional file 1.

Importantly there was a non-adjacent grammatical dependency between the auxiliary and the verb suffix, namely the auxiliary può required the infinitive form while the auxiliary sta required the gerund form. Altogether, 128 different correct sentences were generated (for examples, see Table 1, (1) and (2)).

Incorrect sentences were produced by combining auxiliaries with the incorrectly suffixed verbs from a different sentence (for examples, see Table 1, (3) and (4)). This was done by a cross-splicing procedure at the beginning of each verb. In each sentence the verb was thus exchanged with a verb from a different sentence. To control for splicing effects across conditions, correct sentences were spliced in the same manner. The sentences (1) to (4) are examples from the stimulus material. Incorrect examples are marked with asterisks. All sentences were digitally recorded using a female native speaker of Italian. For each subject, 96 correct sentences were chosen for the learning phases while 32 sentences and their incorrect counterparts were chosen for the testing phases. In each learning phase 64 sentences were presented (256 in all 4 learning phases). Each testing phase comprised 8 correct and 8 incorrect sentences (64 sentences across all testing phases). Importantly, each testing phase contained different auxiliary-verb-suffix triplets than the preceding learning phase in order to ensure that participants learned non-adjacent dependencies (and not auxiliary-verb-suffix trigrams).

### Procedure

**Figure 3:**
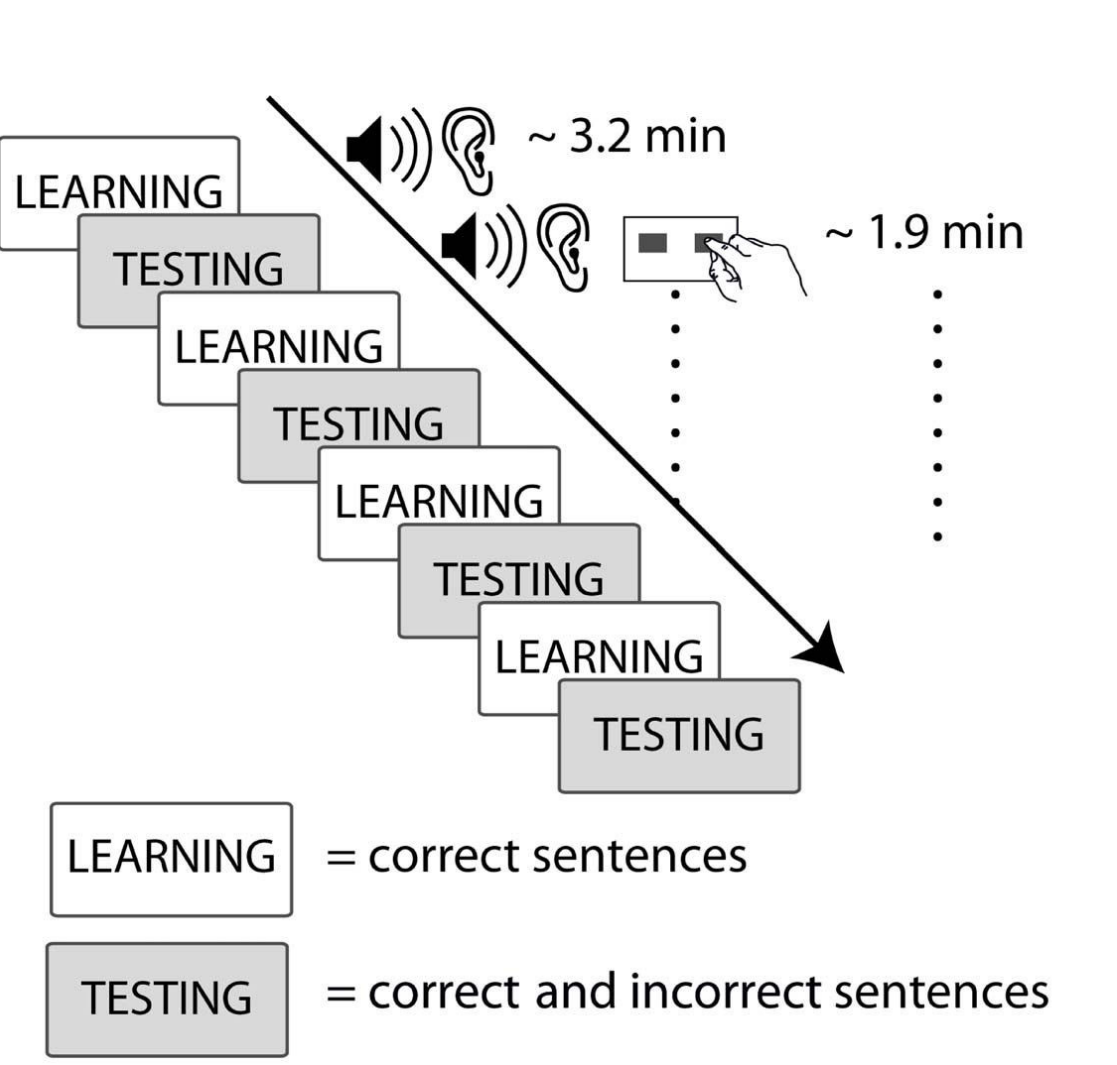
**Procedure**. This figure illustrates the sequence of blocks in the experimental procedure.

Participants were instructed to listen attentively during learning phases and to perform a grammaticality judgment task during the testing phases. During the ERP experiment participants sat in a soundproof booth. Sentences were presented via loudspeakers. The experiment consisted of 4 alternate learning and testing phases starting with a learning phase (see Figure 3). During learning phases participants continuously saw a fixation cross in the middle of a screen placed in front of them. From the beginning of each example sentence to the beginning of the next sentence there was an inter-stimulus-interval (ISI) of 3000 ms. During testing phases the trials started with a fixation cross appearing in the middle of the screen for 1000 ms. Then the test sentence was presented and 3000 ms after the end of the sentence a happy and a sad face appeared on the screen and participants had 2000 ms in which to give a grammaticality judgment. To do this, participants used a 3-button response box (the middle button of which was not used) with the left button corresponding to the 'correct' judgment for 50% of the participants, and the right button for the other 50%. The experiment was conducted without a rest period and there were no pauses between learning and testing phases. The duration of the whole experiment was 24 minutes.

### Data recording and analysis

The EEG was recorded from 59 Ag/AgCl electrodes mounted in an elastic cap (Electro Cap International). The vertical electro-oculogram (EOG) was recorded from two electrodes placed above and below the right eye. The horizontal EOG was measured by electrodes placed at the outer canthus of each eye. During recording, the EEG was referenced to the left mastoid and rereferenced afterwards to the linked mastoids.

Electrode impedances were kept below 5 kΩ and sampling rate was 500 Hz. Trials containing artifacts due to eye movements, muscular activity or amplifier saturation were excluded from ERP averaging. For the automatic artifact rejection of blinks and eyemovements we used a sliding window of 200 ms during which epochs with a standard deviation of > 35 *μ*V were rejected. Other artifacts were rejected manually. ERPs were averaged in the time window from -200 to 2000 ms, time-locked to the beginning of the verb. The epoch from -200 to 0 ms relative to stimulus onset was taken as an amplitude baseline.

For all statistical analyses, the SAS 8.2 software package was used. Due to the different sample sizes and the differences in the general waveform, separate ANOVAs were calculated for the Italian native speaker group and the L2 learner group. We chose two time windows for statistical analysis by visually inspecting the ERP waveforms for each group of participants. In order to assess topographic differences of the ERPs, lateral electrodes were averaged within four regions of interest (ROIs) (left-anterior: Fp1, AF7, AF3, F7, F5, F3, FT7, FC5, FC3, F9, FT9; right-anterior: Fp2, AF8, AF4, F8, F6, F4, FT8, FC6, FC4, F10, FT10; left-posterior: TP7, CP5, CP3, P7, P5, P3, PO7, PO3, O1, TP9, P9; right-posterior: TP8, CP6, CP4, P8, P6, P4, PO8, PO4, O2, TP10, P10). The data were submitted to an ANOVA including the within-subjects factors CORR (incorrect vs. correct), HEM (left hemisphere vs. right hemisphere) and REG (anterior region vs. posterior region). Midline electrodes were analyzed in a separate ANOVA with the factors CORR and REG (anterior midline: Fpz, AFz, Fz, FCz; posterior midline: CPz, Pz, POz, Oz). Further analyses were calculated according to a hierarchical decision criterion. When the global ANOVA revealed at least a marginally significant interaction (p < .10) including the factor CORR, additional ANOVAS were calculated to test which effects on the lower levels were driving the interaction. Only significant main effects including the factor CORR are reported here. All statistical analyses were carried out on unfiltered data. A 7 Hz low pass filter was used for visualization only.

## Authors' contributions

RO and JLM were involved in conception, design, data acquisition and evaluation and interpretation of the study. ADF made substantial contributions to the conception, design and interpretation. All authors contributed to the preparation of the manuscript and read and approved the final version.

## Supplementary Material

Additional file 1 **verblist**. Complete list of the verbs used in the experiment in their infinitive and gerund form. Click here for file
