# Supplementary material for: Syntactic learning by mere exposure - An ERP study in adult learners
Source: BMC Neurosci. 2009 Jul 29;10:89. doi: 10.1186/1471-2202-10-89 (PMC2726980; doi:10.1186/1471-2202-10-89)
Supplement: Additional file 1 — verblist. Complete list of the verbs used in the experiment in their infinitive and gerund form. [file 1471-2202-10-89-S1.pdf]

Complete list of the verbs in their infinitive and gerund form

infinitive form	gerund form
amare	amando
andare	andando
bagnare	bagnando
ballare	ballando
bussare	bussando
cantare	cantando
cercare	cercando
chiamare	chiamando
cullare	cullando
danzare	danzando
entrare	entrando
filmare	filmando
fischiare	fischiando
gelare	gelando
gettare	gettando
giocare	giocando
girare	girando
graffiare	graffiando
gridare	gridando
lodare	lodando
mangiare	mangiando
mostrare	mostrando
ornare	ornando
pagare	pagando
pappare	pappando
passare	passando
pensare	pensando
picchiare	picchiando
stirare	stirando
suonare	suonando
tirare	tirando
volare	volando
